# Interdisciplinary collaboration in traumatic brain injury rehabilitation: a cross-sectional survey of rehabilitation services

**DOI:** 10.3389/fneur.2026.1891136

**Published:** 2026-09-17

**Authors:** Nisreen Al Awaji, Sumayah Aleid, Hussa Alhamin, Sarah Alwarafi, Joud Allhayani, Aisha Alsaban, Danah Al Alsheikh, Maha Aldera

**Affiliations:** 1 Department of Health Communication Sciences, College of Health and Rehabilitation Sciences, Princess Nourah bint Abdulrahman University, Riyadh, Saudi Arabia; 2 Department of Rehabilitation Sciences, College of Health and Rehabilitation Sciences, Princess Nourah bint Abdulrahman University, Riyadh, Saudi Arabia

**Keywords:** healthcare professionals, interdisciplinary collaboration, neurorehabilitation, occupational therapy, rehabilitation, Saudi Arabia, speech-language pathology, traumatic brain injury

## Abstract

**Background:**

Traumatic brain injury (TBI) is a leading cause of mortality and long-term disability worldwide, requiring coordinated rehabilitation services to address complex and multidimensional needs. Despite widespread endorsement of interdisciplinary rehabilitation models, evidence on how such collaboration is implemented in real-world healthcare settings remains limited. This study examined healthcare professionals’ perceptions and experiences of interdisciplinary collaboration in adult TBI rehabilitation in Saudi Arabia.

**Methods:**

A cross-sectional survey was conducted using a convenience sample of 314 healthcare professionals, including physiotherapists (PTs), occupational therapists (OTs), and speech-language pathologists (SLPs) working in rehabilitation settings in Saudi Arabia. The survey assessed professional characteristics, experience with TBI rehabilitation, perceived importance of interdisciplinary practice, collaboration frequency, training exposure, and referral authority. Data were analyzed using descriptive statistics, chi-square tests, one-way analysis of variance with post-hoc comparisons, independent-samples *t*-tests, and correlation analyses.

**Results:**

Responses to the perceived-importance item were mixed: 157 respondents (50.0%) strongly agreed, and 42 (13.4%) agreed that an interdisciplinary approach is important in adult TBI rehabilitation; 63 (20.1%) were neutral, and 52 (16.6%) disagreed or strongly disagreed. The combined agreement figure was 63.4% (199/314). Perceived importance differed significantly by profession, *F*(2, 311) = 10.10, *p* < 0.001, with OTs reporting higher ratings than PTs and SLPs. Collaboration frequency also differed significantly by profession, χ^2^(8, *N* = 314) = 25.15, *p* = 0.0015. Training status was not significantly associated with collaboration frequency, χ^2^(4, *N* = 314) = 8.28, *p* = 0.0819, but was significantly associated with perceived importance, with respondents without prior training reporting higher perceived importance scores, *t*(312) = −2.68, *p* = 0.0077. Age was negatively associated with perceived importance, ρ = −0.3885, *p* < 0.001.

**Conclusion:**

Healthcare professionals demonstrated generally positive attitudes toward interdisciplinary TBI rehabilitation; however, profession-specific differences were observed in both perceived importance and reported collaboration frequency. Training status was not associated with more frequent collaboration, suggesting that education alone may be insufficient without workplace systems that support interdisciplinary practice. These findings highlight the need for structured referral pathways, clearer professional roles, and targeted professional-development initiatives to strengthen interdisciplinary rehabilitation for individuals with TBI.

## 1 Introduction

Neurological disorders comprise a broad category of conditions affecting the central and peripheral nervous systems and can result in substantial functional limitations (1). Traumatic brain injury (TBI) is an acquired brain injury resulting from an external force (2) and is associated with substantial mortality and long-term disability worldwide (3, 4). An estimated 69 million people sustain TBI each year globally (3). Road traffic injuries remain an important cause of TBI, particularly in low-and- middle-income countries (3, 4).

Hospital-based studies provide important local context for the burden of adult TBI in Saudi Arabia. In a retrospective study of 423 adults with acute TBI treated at a tertiary medical center in Jeddah, road traffic accidents were the most common injury mechanism (49.7%), and 7.0% of patients died in hospital (5). Although these single-center findings should not be interpreted as national estimates of incidence or mortality, they illustrate the clinical importance of TBI within the Saudi context.

Adult TBI can produce concurrent physical, cognitive, communication, behavioral, and emotional impairments that affect functioning and participation (6). These multidimensional rehabilitation needs may require coordinated input from PTs, OTs, and SLPs. In this context, interdisciplinary practice involves professionals from different disciplines working toward shared goals, coordinating decisions, and sharing responsibility for care (7). Such coordination may reduce fragmented treatment and help ensure that rehabilitation plans address needs that extend beyond the scope of any single discipline (8, 9).

Published reports from Riyadh illustrate that adult TBI rehabilitation is delivered through both dedicated inpatient rehabilitation and acute hospital-based programmes. A specialized inpatient TBI unit has described coordinated care involving physicians, nurses, PTs, OTs, SLPs, psychologists, case managers, nutritionists, patients, and families (10). At a major trauma center, a structured acute intensive trauma rehabilitation programme was developed in coordination with physical medicine and rehabilitation, physiotherapy, occupational therapy, speech-language pathology, prosthetics and orthotics, and social work after earlier therapy services had not been fully integrated into patient care (11). These reports also describe constraints in rehabilitation capacity, service integration, and timely access, while a national review identifies access, workforce allocation, and service coordination as continuing priorities for rehabilitation development in Saudi Arabia (10–13).

Although interdisciplinary rehabilitation is widely recommended for traumatic brain injury, evidence on how such collaboration is implemented in real-world service settings in Saudi Arabia and the wider Middle East and North Africa (MENA) region remains limited. Existing Saudi studies have described outcomes from individual inpatient rehabilitation programmes, and a regional systematic review identified a scarcity of TBI research across MENA (10, 12, 14). Understanding healthcare professionals’ perspectives on interdisciplinary collaboration can provide important insights into organzational and system-level factors that influence integrated rehabilitation care. Accordingly, this study examined the perceptions and experiences of PTs, OTs, and SLPs involved in adult TBI rehabilitation in Saudi Arabia. Perspectives of other members of the broader interdisciplinary rehabilitation team were outside the scope of this study. The study addresses the following research question: What are the perceptions and experiences of PTs, OTs, and SLPs regarding interdisciplinary collaboration in adult traumatic brain injury rehabilitation in Saudi Arabia?

## 2 Methods

### 2.1 Study design

A cross-sectional study design was employed to collect data from healthcare professionals involved in TBI rehabilitation in Saudi Arabia. A quantitative survey was developed and distributed to gather information on their perceptions and practices related to interdisciplinary collaboration.

### 2.2 Participants and recruitment

The study included a convenience sample of 314 healthcare professionals after excluding 14 respondents with non-standard professional classification entries. Eligibility was restricted to respondents who self-reported a professional classification of Specialist, Senior Specialist, or Consultant. These titles correspond to professional classification categories used by the Saudi Commission for Health Specialists (SCFHS), which evaluates submitted qualifications, speciality-specific training, professional registration, professional experience where applicable, and professional assessment when required (15). Although these categories broadly denote professional progression, their precise qualification and experience thresholds vary across health disciplines; they should therefore be interpreted as indicators of regulatory professional rank rather than fixed years-in-practice categories. Years of professional experience were reported separately to describe the experience profile of the sample. Professional classification and the separate referral-authority item were treated as distinct measures; classification was not interpreted as conferring uniform referral rights. Participants were recruited from various healthcare settings, including public and private hospitals, as well as specialized rehabilitation centers across Saudi Arabia. Recruitment was conducted via professional networks and social media channels. Due to the nature of convenience sampling, a formal response rate could not be calculated.

### 2.3 Survey instrument

A structured online survey was investigator-developed to assess multiple dimensions of interdisciplinary practice. Item domains were selected to capture participant characteristics, clinical experience with TBI rehabilitation, perceptions and reported practices of interdisciplinary collaboration, training and education, perceived functional benefit, and future perspectives. Before distribution, the draft questionnaire underwent an informal review by three rehabilitation practitioners: one OT, one SLP, and one PT, to assess wording and clarity. Minor refinements were made before distribution. The instrument was not formally validated; therefore, findings derived from the survey items should be interpreted as exploratory. The survey was divided into several sections:

- Demographic information: Age, gender, occupation, professional classification, nationality, workplace, and years of experience.
- Experience with TBI: Frequency of treating TBI patients and familiarity with the impact of TBI on motor and communication skills.
- Perceptions and practices: Individual items assessing familiarity with other disciplines, agreement with the importance of an interdisciplinary approach, frequency of collaboration, and confidence in referring patients.
- Training and education: Items assessing previous training in interdisciplinary practice and perceived need for such training.
- Functional improvement: An item assessing the perceived effectiveness of the interdisciplinary approach.
- Future perspectives: An item assessing the likelihood of recommending initiatives to enhance interdisciplinary practice.

Response formats were specified as follows. Perceived importance of an interdisciplinary approach in adult TBP rehabilitation was rated on a five-point Likert-type scale: Strongly disagree, Disagree, Neutral, Agree, and Strongly agree. The full five-category response was retained for analysis and was not dichotomized. For descriptive reporting only, “Agree or Strongly agree” denotes the combined Agree and Strongly agree categories; this combined result was 199 of 314 respondents (63.4%), while the separate-category distribution is reported in the Results. Collaboration frequency was rated as Always, Frequently, Sometimes, Rarely, or Never. Familiarity with the impact of TBI and with other disciplines’ roles was rated as Very familiar, Moderately familiar, Somewhat familiar, or Not familiar; role-familiarity items also included a profession-specific “Not applicable” option.

Confidence in identifying the need for referral was rated as Not at all confident, Slightly confident, Moderately confident, Very confident, or Extremely confident. Referral authority and prior interdisciplinary-practice training used Yes/No responses, and team communication was rated as Excellent, Good, Fair, or Poor. The perceived need for training was rated as Not at all, Slightly, Moderately, Very much, or Extremely; perceived effectiveness was rated as Not effective at all, Somewhat effective, Effective, Very effective, or Unsure; likelihood of recommending initiatives was rated as Very likely, Moderately likely, Slightly likely, or Not likely; and the current state of interdisciplinary practice was rated as Very unsatisfying, Unsatisfying, Neutral, Satisfying, or Very satisfying. Other items used categorical, free-text, or select-all-that-apply response formats. The complete questionnaire, including exact item wording and response options, is provided in Supplementary file 1.

Referral authority was assessed by asking whether respondents had authority in their workplace to refer patients to other specialists based on patient needs. In this study, the item captured self-reported, workplace-specific authority to refer within the respondent’s rehabilitation setting; it did not establish statutory scope of practice, prescribing authority, or a uniform national referral entitlement.

### 2.4 Data analysis

Descriptive statistics were used to analyze the collected data. Frequencies and percentages were calculated for categorical variables, while means and standard deviations were calculated for continuous variables. A total of 328 submitted responses were identified. Fourteen responses with non-standard professional-classification entries were excluded to maintain consistency in the analytic professional-classification groups, resulting in a final analytic sample of 314 respondents. Age was available as a valid numeric response for 301 participants; 13 entries were missing or non-numeric. Years of professional experience were available as a valid numeric response for 293 participants; 21 entries were missing or non-numeric. Missing or non-numeric values were treated as missing and were not imputed. Descriptive statistics for age and professional experience were calculated using available valid numeric responses. The Spearman correlation between age and perceived importance used respondents with valid values for both variables.

To examine relationships between variables, the following inferential statistical tests were performed:

- Chi-square tests of independence were used to assess associations between categorical variables. Cramér’s V was reported as an effect-size measure where appropriate.
- One-way analysis of variance (ANOVA) was used to compare mean perceived-importance scores across professional groups. Tukey post-hoc comparisons were conducted for pairwise group comparisons, and eta-squared was reported as an effect-size measure.
- Independent-samples *t*-tests were used to compare mean perceived-importance scores by training status. Cohen’s d was reported as an effect-size measure.
- Spearman’s rank correlation was used to examine the association between age and perceived importance.
- Kruskal–Wallis testing was conducted as a non-parametric sensitivity analysis for profession-level differences in perceived importance.

Statistical significance was set at *p* < 0.05 for all tests. The analysis was performed using Python 3.11 with the pandas, scipy, and statsmodels libraries.

## 3 Results

### 3.1 Demographic characteristics of respondents

A total of 328 submitted responses were identified in the dataset. After excluding 14 respondents whose professional classification did not correspond to Specialist, Senior Specialist, or Consultant, the final analytic sample comprised 314 healthcare professionals. The mean age was 31.5 years (SD = 7.5; valid *n* = 301; 13 entries missing or non-numeric), and the sample was slightly more female than male (51.9% female; 48.1% male). The sample included 111 SLPs (35.4%), 107 OTs (34.1%), and 96 PTs (30.6%). Most respondents were Saudi nationals (94.6%). Regarding workplace setting, 43.9% worked in private hospitals or clinics, 29.0% in public hospitals, 22.6% in specialized centers, and 4.5% in other settings. The mean professional experience was 6.3 years (SD = 4.7; valid *n* = 293; 21 entries missing or non-numeric). Participant demographic characteristics are presented in Table 1.

**Table 1:** Demographic characteristics of respondents (*N* = 314).

Characteristic	Category	Frequency (*n*)	Percentage (%)
Age (Mean ± SD)	31.5 ± 7.5 years	-	-
Gender	Female	163	51.9
	Male	151	48.1
Occupation	OT	107	34.1
	SLP	111	35.4
	PT	96	30.6
Professional classification	Specialist	182	58.0
	Senior Specialist	112	35.7
	Consultant	20	6.4
Nationality	Saudi	297	94.6
	Non-Saudi	17	5.4
Workplace	Private hospital/clinic	138	43.9
	Public hospital	91	29.0
	Specialized center	71	22.6
	Other	14	4.5
Experience (Mean ± SD)	6.3 ± 4.7 years	-	-

Percentages are based on the final analytic sample (*N* = 314) unless otherwise indicated. Age was calculated using valid numeric responses (*n* = 301; 13 missing or non-numeric entries), and years of professional experience were calculated using valid numeric responses (*n* = 293; 21 missing or non-numeric entries). Missing or non-numeric values were excluded from the relevant analyses and were not imputed. OT, occupational therapist; PT, physiotherapist; SLP, speech-language pathologist; SD, standard deviation. Professional classification was self-reported and reflects SCFHS regulatory professional rank rather than a uniform years-in-practice threshold.

### 3.2 Perceptions of interdisciplinary practice

#### 3.2.1 Perceived importance of interdisciplinary approach

Responses to the perceived-importance item were distributed across the full five-point scale: 157 respondents (50.0%) strongly agreed and 42 (13.4%) agreed that an interdisciplinary approach is important in adult TBI rehabilitation; 63 (20.1%) were neutral, 27 (8.6%) disagreed, and 25 (8.0%) strongly disagreed. For descriptive context, the combined Agree-or-Strongly-agree category included 199 of 314 respondents (63.4%; Wilson 95% CI: 57.9–68.5%); this summary did not replace the full-scale analysis. Perceived importance differed significantly by profession, *F*(2, 311) = 10.10, *p* = 0.000056, η^2^ = 0.061. OTs reported the highest perceived importance score (mean = 4.34, SD = 1.24), followed by PTs (mean = 3.73, SD = 1.46) and SLPs (mean = 3.59, SD = 1.17). Tukey *post-hoc* comparisons showed that OTs rated the importance of interdisciplinary practice significantly higher than PTs (adjusted *p* = 0.0026) and SLPs (adjusted *p* = 0.0001). The difference between PTs and SLPs was not statistically significant (adjusted *p* = 0.7339). A Kruskal-Wallis sensitivity analysis yielded the same substantive conclusion, H(2) = 26.81, *p* = 0.001.

#### 3.2.2 Collaboration frequency

Collaboration frequency differed significantly across professions, χ^2^(8, *N* = 314) = 25.15, *p* = 0.0015, Cramér’s V = 0.200. OTs most frequently reported “always” collaborating with other disciplines (47.7%), followed by PTs (35.4%) and SLPs (21.6%). Conversely, SLPs more frequently reported “sometimes” collaborating (28.8%) compared with OTs (18.7%) and PTs (16.7%). Detailed collaboration-frequency distributions by professional group are presented in Table 2.

**Table 2:** Collaboration frequency by profession, *n* = 314.

Profession	Always *n* (%)	Frequently *n* (%)	Sometimes *n* (%)	Rarely *n* (%)	Never *n* (%)
OTs, *n* = 107	51 (47.7)	29 (27.1)	20 (18.7)	7 (6.5)	0 (0.0)
PTs, *n* = 96	34 (35.4)	28 (29.2)	16 (16.7)	12 (12.5)	6 (6.2)
SLPs, *n* = 111	24 (21.6)	38 (34.2)	32 (28.8)	14 (12.6)	3 (2.7)

Percentages are calculated within each professional group. Cramér’s V was used as the effect-size measure for the chi-square analysis.

### 3.3 Training and education

Most respondents reported having received specific training or education in interdisciplinary practice (217/314; 69.1%). The association between training status and collaboration frequency was not statistically significant at the conventional 0.05 level, χ^2^(4, *N* = 314) = 8.28, *p* = 0.0819, Cramér’s V = 0.162. However, perceived importance differed by training status. Respondents without prior training reported higher perceived importance scores (mean = 4.19, SD = 1.28) than those with prior training (mean = 3.76, SD = 1.33), *t*(312) = −2.68, *p* = 0.0077, Cohen’s d = −0.328. In exploratory descriptive analyses, the untrained group was younger on average than the trained group (mean age = 28.17 vs. 33.02 years). Higher perceived-importance scores among untrained respondents were also observed within each profession (PTs: 4.11 vs. 3.48; OTs: 4.46 vs. 4.29; SLPs: 4.03 vs. 3.42). These *post hoc* comparisons were not adjusted for potential confounders and are reported only to contextualize the association.

### 3.4 Other factors

Age was significantly and negatively correlated with perceived importance of interdisciplinary practice, Spearman ρ = −0.3885, *p* = 2.77 × 10^-12, valid *n* = 301, indicating that older respondents tended to report lower perceived importance scores. Professional classification was significantly associated with self-reported workplace authority to refer patients to other specialists, χ^2^(2, N = 314) = 6.55, *p* = 0.0377, Cramér’s V = 0.145, although the effect size was small. Workplace type was not significantly associated with collaboration frequency after consolidating workplace categories into private hospital/clinic, public hospital, specialized center, and other settings, χ^2^(12, *N* = 314) = 20.30, *p* = 0.0616, Cramér’s V = 0.147.

## 4 Discussion

This study provides empirical evidence on healthcare professionals’ perceptions and reported practices of interdisciplinary collaboration in adult TBI rehabilitation in Saudi Arabia. Specifically, it identifies profession-specific differences in perceived importance and reported collaboration frequency, alongside associations with training status, age, and self-reported workplace referral authority. On the perceived-importance item, 157 respondents (50.0%) strongly agreed, and 42 (13.4%) agreed that an interdisciplinary approach is important in adult TBI rehabilitation; 63 (20.1%) were neutral, and 52 (16.6%) disagreed or strongly disagreed. The 63.4% figure is a descriptive combination of the Agree and Strongly agree categories and does not represent a dichotomized inferential analysis. This response pattern should be interpreted with the full distribution in view. This finding aligns with a growing body of international literature advocating for integrated care models to address the complex needs of TBI survivors (8, 9). However, the results also show important profession-specific differences and several nuanced associations that require careful interpretation. Against this established context, the following interpretation focuses on how the observed profession-specific patterns and organizational factors may shape interdisciplinary collaboration in adult TBI rehabilitation in Saudi Arabia.

A key finding of the current study is the significant variation in perceived importance of interdisciplinary practice across professions. OTs reported the highest perceived importance scores, followed by physiotherapists and SLPs. This difference was statistically significant and remained significant in a non-parametric sensitivity analysis, although the effect size should be interpreted as small-to-moderate rather than large. The higher ratings among occupational therapists may be attributable to the holistic and client-centered philosophy that underpins OT, which naturally aligns with the principles of interdisciplinary collaboration (16).

The findings also indicate significant profession-specific differences in collaboration frequency. OTs most frequently reported “always” collaborating with other disciplines, whereas SLPs reported the lowest proportion of “always” collaborating and a higher proportion of “sometimes” collaborating. This finding should be interpreted carefully. It does not necessarily indicate lower willingness or professional commitment among SLPs; rather, it may reflect structural, environmental, and organizational factors that shape opportunities for collaboration.

One possible explanation concerns the organization of SLP service delivery in TBI rehabilitation. The physical and acoustic environment can influence communication accessibility for people with communication disabilities (17). In some rehabilitation settings, attention to communication accessibility may be associated with the use of separate treatment spaces; this could reduce opportunities for informal, spontaneous interaction with other professionals. However, treatment location and environmental conditions were not measured in the present study, and this proposed mechanism should therefore be interpreted cautiously. Environmental and contextual barriers to SLP service delivery have been identified in other healthcare settings (18); however, their specific contribution to collaboration patterns in adult TBI rehabilitation warrants further investigation.

Beyond environmental factors, role clarity and service-integration issues may also contribute to profession-specific collaboration patterns. The role of SLPs in TBI rehabilitation, especially in cognitive-communication rehabilitation beyond aphasia and dysphagia, may not always be fully understood by other professionals. This could lead to fewer referrals, delayed involvement, or less integration into the core rehabilitation team (8, 19). Studies have also identified role confusion as a barrier to effective team functioning in rehabilitation settings (20, 21). In addition, scheduling complexity, separate documentation systems, limited staffing, and lack of dedicated time for team meetings may create logistical barriers to collaboration (19). Therefore, the lower reported collaboration frequency among SLPs should be understood in relation to service structures rather than interpreted as an individual-level deficit.

The study also uncovered a paradoxical relationship with training. Although most respondents had received specific training or education in interdisciplinary practice, training status was not significantly associated with collaboration frequency. Therefore, the findings do not support the conclusion that trained professionals collaborate significantly more frequently than untrained professionals. Nevertheless, training status was significantly associated with perceived importance, with respondents without prior training reporting higher perceived importance scores than those with training. This counterintuitive finding should be interpreted cautiously. It may reflect ceiling effects in ratings of perceived importance; differences in professional role or workplace exposure; or self-selection into training. It is also possible that training exposes professionals to the practical challenges and limitations of interdisciplinary work, resulting in more realistic or critical appraisals. Alternatively, existing training programs may emphasize procedural aspects of collaboration without sufficiently reinforcing the theoretical rationale and patient-centered benefits of interdisciplinary practice. Further research is needed to examine the content, quality, duration, and outcomes of interdisciplinary training programs. The available data cannot determine whether this reflects idealized perceptions among untrained respondents, greater exposure to implementation challenges among trained respondents, or age- and role-related differences. Exploratory descriptive comparisons showed that untrained respondents were younger on average and that their higher perceived-importance scores occurred within each profession; however, these comparisons were not adjusted and do not establish any underlying mechanism.

The study also showed a significant negative association between age and perceived importance of interdisciplinary practice. This suggests that younger professionals tended to report stronger endorsement of interdisciplinary rehabilitation. This may reflect generational differences in professional education, as contemporary health-professions curricula increasingly emphasize interprofessional learning and collaborative practice (7). However, as the study is cross-sectional, this association should not be interpreted as evidence of a causal generational shift. It may also reflect differences in workplace exposure, professional role expectations, or career-stage experiences.

Another finding concerns professional classification and self-reported workplace authority to refer patients to other specialists. The results showed a statistically significant association between professional classification and authority to refer patients to other specialists, although the effect size was small. The survey item did not determine statutory scope of practice, prescribing authority, or facility-specific referral policies; therefore, the association should not be interpreted as showing that SCFHS classification itself confers referral rights. Rather, it indicates that respondents’ reported authority to make a cross-disciplinary referral differed modestly across the professional-classification groups represented in this sample. From a practice perspective, this finding is important because interdisciplinary rehabilitation depends not only on positive attitudes but also on institutional systems that permit timely referral and cross-disciplinary access. Clear facility-level referral pathways and role-specific protocols may therefore be needed to support effective interdisciplinary care.

These findings should also be interpreted in relation to the organization of rehabilitation services in Saudi Arabia. Published reports from Riyadh describe both a dedicated inpatient TBI rehabilitation unit and an acute hospital-based trauma rehabilitation programme that integrate PTs, OTs, SLPs, physicians, nurses, and other disciplines, while also identifying constraints in rehabilitation capacity, service integration, timely access, workforce allocation, and care coordination (10–13). These reports do not establish the conditions at all facilities represented in the present sample. Nevertheless, the profession-specific differences in reported collaboration and self-reported workplace referral authority may reflect variation in how rehabilitation teams are staffed, organized, and linked through local referral workflows. As facility-level team composition, service capacity, and referral policies were not measured, this remains a contextual interpretation that should be examined in future multisite research.

Workplace type was not significantly associated with collaboration frequency after consolidating workplace categories into private hospital/clinic, public hospital, specialized center, and other settings. Although the association approached conventional statistical significance, this finding should be interpreted as inconclusive. This suggests that collaboration barriers may not be limited to a single type of workplace but may instead reflect broader organizational and system-level challenges across rehabilitation settings. Future studies with larger and more balanced samples across workplace categories may be better positioned to examine whether specific institutional settings facilitate or hinder interdisciplinary collaboration.

### 4.1 Implications for practice and policy

Given the cross-sectional design, convenience sample, self-reported measures, and absence of formal psychometric validation, the following implications should be interpreted as context-specific hypotheses and priorities for future research or service evaluation rather than direct evidence of effectiveness, causation, or prescriptive policy conclusions. The findings nonetheless identify areas that may merit closer examination in adult TBI rehabilitation settings in Saudi Arabia.

First, the profession-specific variation in perceived importance and collaboration frequency may warrant further investigation of context-specific barriers and facilitators. Future research or service evaluation could examine whether approaches such as clearer referral pathways, shared care planning, and protected time for team communication are associated with more integrated participation of SLPs in adult TBI rehabilitation teams. The present data do not establish the effectiveness of any such approach.

Second, the training findings raise hypotheses that could be examined in future research. Although training status was not significantly associated with collaboration frequency in this sample, this result does not establish that training is ineffective or insufficient. Future studies and service evaluations could examine whether training approaches that are paired with practical workplace mechanisms, such as structured case conferences, shared documentation systems, standardized communication protocols, and clearly defined team roles, are associated with changes in collaborative practice. The higher perceived-importance scores reported by respondents without prior training also warrant cautious investigation; possible explanations include differences in training content, professional role, workplace exposure, or self-selection, none of which can be determined from the present data.

Third, the modest association between professional classification and self-reported workplace referral authority may justify facility-level examination of referral processes in future work. It does not show that professional classification determines referral rights or that particular policy changes would improve care. Rehabilitation institutions could consider evaluating whether explicit, role-specific referral protocols improve the timeliness and coordination of cross-disciplinary care. Such evaluation may be relevant in TBI rehabilitation, where patients can require input from physiotherapy, occupational therapy, speech-language therapy, psychology, social work, nursing, and medical specialists.

Fourth, the negative association between age and perceived importance warrants further investigation rather than direct adaptation of educational and professional-development strategies. Future research could examine whether this association reflects career stage, professional role, workplace exposure, prior interprofessional education, or other unmeasured factors. If future studies corroborate such differences, context- and career-stage-sensitive approaches to professional development could then be evaluated.

### 4.2 Limitations

This study has several limitations that should be considered when interpreting the findings. First, the use of a convenience sample may introduce selection bias, as participants may not be representative of all healthcare professionals involved in TBI rehabilitation in Saudi Arabia. Second, the analytic sample was restricted to respondents with standard professional classifications, which may limit generalizability to interns, students, faculty members, and other non-standard professional groups. Third, the investigator-developed survey underwent only an informal review by three rehabilitation practitioners and was neither pilot-tested nor formally assessed for face/content validity or psychometric properties. The findings should therefore be interpreted as exploratory, and future research should develop and validate a contextually appropriate instrument. Fourth, some variables, such as age and years of professional experience, had missing or non-numeric responses, resulting in lower valid denominators for those descriptive statistics. Fifth, the cross-sectional design does not allow causal inferences to be made. Associations between training, age, profession, and perceptions should therefore be interpreted as correlational rather than causal.

Finally, the study did not include other key members of interdisciplinary rehabilitation teams, such as neuropsychologists, rehabilitation physicians, nurses, social workers, and case managers, who may have provided a more comprehensive understanding of interdisciplinary practice. Recruitment through professional networks and social-media platforms may also have contributed to selection bias, as professionals active on these channels may differ systematically from the broader population of TBI rehabilitation professionals in Saudi Arabia. In addition, the Saudi-specific professional-classification and workplace referral context may limit the transferability of these findings to healthcare systems with different regulatory frameworks, scopes of practice, rehabilitation-service structures, or referral processes. This restriction may have underrepresented the perspectives of professionals who influence medical decision-making, referral pathways, psychosocial support, and case coordination. As formal reliability assessment was not undertaken, the measurement consistency and precision of the survey responses cannot be established.

## 5 Conclusion

Healthcare professionals in Saudi Arabia reported mixed perceptions of interdisciplinary practice in TBI rehabilitation: 157 respondents (50.0%) strongly agreed, and 42 (13.4%) agreed that an interdisciplinary approach is important, whereas 63 (20.1%) were neutral and 52 (16.6%) disagreed or strongly disagreed. The study identified profession-specific differences in both perceived importance and reported collaboration frequency; OTs reported the highest perceived importance and the highest proportion of “always” collaborating, whereas SLPs reported less frequent collaboration. These patterns do not establish their underlying causes but may warrant further investigation of service organization, role clarity, referral workflows, and workplace communication in adult TBI rehabilitation. Training status was not significantly associated with collaboration frequency; future research should examine how training content and workplace context may relate to collaborative practice. Future research should use validated instruments, more rigorous sampling strategies, and longitudinal or mixed-methods designs to examine how interdisciplinary rehabilitation models influence both professional practice and patient outcomes.

## Data Availability

The raw data supporting the conclusions of this article will be made available by the authors, without undue reservation.
