# Supplementary material for: Interdisciplinary collaboration in traumatic brain injury rehabilitation: a cross-sectional survey of rehabilitation services
Source: Front Neurol. 2026 Sep 17;17:1891136. doi: 10.3389/fneur.2026.1891136 (PMC13626885; doi:10.3389/fneur.2026.1891136)
Supplement: Supplementary file 1 [file Table_1.docx]

**Supplementary File 1**

**Title:** Traumatic Brain Injury Rehabilitation: An Interdisciplinary Approach to Physiotherapy, Speech-Language Pathology, and Occupational Therapy

**Demographic Information:**

- Age:
- …………….
- Gender:
- Male
- Female
- Occupation:
- Physiotherapist
- Speech-Language Pathologist
- Occupational Therapist
- Professional classification:
- Consultant
- Senior specialist
- Specialist
- Other (please specify):
- Nationality:
- Saudi
- Non-Saudi
- Workplace:
- Public hospital
- Private hospital/clinic
- Specialized center
- Other (please specify):
- Place of residence:
- Central
- Western
- Eastern
- Southern
- Northern
- Years of professional experience:
- …………………………………

**Experience:**

- **How frequently do you treat patients diagnosed with traumatic brain injury in your clinic?**
- Never
- 1-5 per year
- 6-10 per year
- More than 10 per year
- **How familiar are you with the impact of traumatic brain injury on motor skills?**
- Very familiar
- Moderately familiar
- Somewhat familiar
- Not familiar
- **How familiar are you with the impact of traumatic brain injury on communication skills?**
- Very familiar
- Moderately familiar
- Somewhat familiar
- Not familiar

**Perception of interdisciplinary practice:**

- **If you are not a physiotherapist, how familiar are you with physiotherapists' role in managing patients with traumatic brain injury?**
- Very familiar
- Moderately familiar
- Somewhat familiar
- Not familiar
- Not applicable, I am a physiotherapist
- **If you are not an occupational therapist, how familiar are you with occupational therapists' role in managing patients with traumatic brain injury?**
- Very familiar
- Moderately familiar
- Somewhat familiar
- Not familiar
- Not applicable, I am an occupational therapist
- **If you are not a speech pathologist, how familiar are you with speech pathologists' role in managing patients with traumatic brain injury?**
- Very familiar
- Moderately familiar
- Somewhat familiar
- Not familiar
- Not applicable, I am a speech pathologist
- **To what extent do you agree with the importance of an interdisciplinary approach in addressing traumatic brain injury in patients?**
- Strongly disagree
- disagree
- Neutral
- Agree
- Strongly agree
- **How often do you collaborate with other disciplines; such as physiotherapists, occupational therapists, and/or speech-language pathologists; in the rehabilitation of patients with TBI?**
- Always
- Frequently
- Sometimes
- Rarely
- Never
- **What specialty do you work in collaboration with? (select all that apply)**
- Physiotherapists
- Occupational therapists
- Speech-language pathologists
- **Please indicate your level of confidence in identifying the necessity of referring a patient to other specialists based on the patient's needs:**
- Not at all confident
- Slightly confident
- Moderately confident
- Very confident
- Extremely confident
- **Do you have the authority in your workplace to refer patients to other specialists based on their needs?**
- Yes
- No
- **Rate the communication skills of the interdisciplinary team members in your place.**
- Excellent
- Good
- Fair
- Poor
- **In your opinion, what are the biggest challenges you face in collaborating with other disciplines in TBI rehabilitation? (select all that apply)**
- Lack of understanding of other disciplines' roles
- Staff shortages
- Limited resources
- Limited training in interdisciplinary practice
- Limited communication channels
- Differing treatment philosophies
- Scheduling conflicts
- Other (Please specify)
- **What resources would be most helpful to you in facilitating effective interdisciplinary collaboration in TBI rehabilitation? (Select all that apply)**
  - Joint training opportunities for different disciplines
  - Standardized communication protocols
  - Shared electronic medical records
  - Clear guidelines for roles and responsibilities of each team member
  - Other (Please specify)
- **What factors hinder your engagement in interdisciplinary practices?** **(select all that apply)**
- Lack of awareness about other professions' duties
- Staff shortages
- Limited resources
- Limited training in interdisciplinary practice
- Other (please specify)
- **In your opinion, what are the advantages of employing an interdisciplinary practice when treating patients with traumatic brain injury?**

**Training and Education:**

- **Have you received any specific training or education on interdisciplinary practice?**
- Yes
- No
- **To what extent do you believe therapists working in the management of patients with traumatic brain injury should receive training in interdisciplinary practice?**
- Not at all
- Slightly
- Moderately
- Very much
- Extremely

**Functional Improvement for Traumatic Brain Injury Rehabilitation:**

- **In your experience, what are the most important goals for TBI rehabilitation? (Select all that apply)**
  - Improving physical function
  - Enhancing cognitive skills (e.g., memory, attention)
  - Maximizing communication abilities
  - Promoting emotional well-being
  - Increasing independence in daily activities
  - Other (Please specify)
- **In your opinion, what are the biggest benefits of an interdisciplinary approach to TBI rehabilitation? (Select all that apply)**
  - Provides a more holistic understanding of patient needs
  - Allows for coordinated treatment goals across disciplines
  - Improves communication and collaboration among providers
  - Leads to better patient outcomes
  - Other (Please specify)
- **In your experience, what are the most improvement you observe in TBI rehabilitation? (Select all that apply)**
  - Physical abilities (e.g., walking, balance) (Yes/No)
  - Communication skills (e.g., speaking, understanding) (Yes/No)
  - Daily living activities (e.g., dressing, bathing) (Yes/No)
  - Overall functional independence (Yes/No)
- **In your experience, how effective is an interdisciplinary approach (involving physiotherapy, speech-language pathology, and occupational therapy) in improving outcomes for adult TBI patients undergoing rehabilitation?**
  - Not effective at all
  - Somewhat effective
  - Effective
  - Very effective
  - Unsure

**Future Perspectives:**

- **What of the following would encourage you to collaborate in an interdisciplinary practice? (select all that apply)**
- Establish interdisciplinary teams
- Clearly define the roles and responsibilities of each team member
- Offer training opportunities
- Involve families as active participants in interdisciplinary team meetings
- Other (please specify)
- **How likely will you recommend specific initiatives to enhance interdisciplinarity in your workplace?**
- Very likely
- Moderately likely
- Slightly likely
- Not likely
- **Rate your perception of the current state of interdisciplinary practice in the context of managing patients with disabilities:**
- Very unsatisfying
- Unsatisfying
- Neutral
- Satisfying
- Very satisfying
